# Deforestation and Malaria in Mâncio Lima County, Brazil

**DOI:** 10.3201/eid1607.091785

**Published:** 2010-07

**Authors:** Sarah H. Olson, Ronald Gangnon, Guilherme Abbad Silveira, Jonathan A. Patz

**Affiliations:** Author affiliations: University of Wisconsin, Madison, Wisconsin, USA (S.H. Olson, R. Gangnon, J.A. Patz);; Santo Antônio Energia, Porto Velho, Brazil (G. Silveira)

**Keywords:** vector-borne infections, malaria, deforestation, Brazil, Anopheles spp., mosquitoes, surveillance, South America, research

## Abstract

Malaria is the most prevalent vector-borne disease in the Amazon. We used malaria reports for health districts collected in 2006 by the Programa Nacional de Controle da Malária to determine whether deforestation is associated with malaria incidence in the county (município) of Mâncio Lima, Acre State, Brazil. Cumulative percent deforestation was calculated for the spatial catchment area of each health district by using 60 × 60–meter, resolution-classified imagery. Statistical associations were identified with univariate and multivariate general additive negative binomial models adjusted for spatial effects. Our cross-sectional study shows malaria incidence across health districts in 2006 is positively associated with greater changes in percentage of cumulative deforestation within respective health districts. After adjusting for access to care, health district size, and spatial trends, we show that a 4.3%, or 1 SD, change in deforestation from August 1997 through August 2000 is associated with a 48% increase of malaria incidence.

## Abstract 2

Deforestation is associated with elevated risk for malaria in the Amazon.

Malaria risk in the Amazon and around the malaria belt is an integrated mix of environmental and sociodemographic risk factors (*1*–*3*). Despite >50 years of malaria control efforts from 1997 through 2006, on average, Brazil had ≈500,000 confirmed cases annually (*4*,*5*). Most malaria cases in Brazil occur in the Amazon Basin, where logging rates between 1999 and 2002 ranged from 12,000 to 20,000 km^2^ per year, the sum of which would cover the country of Denmark (*6*).

The main vectors of malaria in the Amazon, *Anopheles darlingi* mosquitoes, seek out larval habitat in partially sunlit areas, with clear water of neutral pH and aquatic plant growth, and they are notably present and more abundant in altered landscapes (*7*–*9*). In Peru, *A. darlingi* mosquitoes are seldom observed in standing water bodies within undisturbed forests because they are shaded and soils are more acidic, and yet these forests remain abundant and rich in mosquito species that do not transmit malaria (*9*,*10*). Along the Iquitos–Nauta Road corridor entomologic risk factors of mosquito biting rate and larval count increase with more deforestation. The mean biting rate in areas with >80% deforestation was 8.33 compared with 0.03 per night for sites with <30% deforestation (*10*). Furthermore, the likelihood of finding *A. darlingi* larvae doubled in breeding sites with <20% forest compared with sites with 20%–60% forest, and the likelihood increased 7-fold when compared with sites with >60% forest (*8*). Human-altered landscapes provide a milieu of suitable larval habitats for *A. darling* mosquitoes, including road ditches, dams, mining pits, culverts, vehicle ruts, and areas of poor clearing.

The characteristics of these mosquitoes’ preferred habitat and studies of human and entomologic malaria risk suggest that deforestation and land clearing contribute to the dynamic malaria patterns along the frontier of settlement. Frontier malaria theory explains this pattern in new settlements as follows: an initial epidemic occurs that abates to persistent low incidence and eventually eradication as the result of changing social, ecologic, and environmental relationships (*11*). For instance, from 1985 through 1995, malaria risk in Rondônia increased during the initial colonization phase due to ecosystem transformations that promoted larval habitats and then gradually subsided as urban area expanded, agriculture became established, settlers became more knowledgeable, access to healthcare increased, home construction improved, and suitable larval habitats declined, until, finally, malaria risk was mostly linked to human behavioral factors (*2*,*12*). Frontier malaria theory is further supported by research around the Granada area in Acre, where a population-based cohort study found land clearing activities and <5 years of residence associated with higher probability of PCR-confirmed malaria morbidity (*13*).

In this 2006 cross-sectional study, we examined the association of deforestation, socioeconomic and demographic factors, and malaria at the level of health districts (*localidades*) using a uniform surveillance tool implemented in 2003 by the Brazilian Ministry of Health's Programa Nacional de Controle da Malária (PNCM). This nationally standardized system covers 5.1 million km^2^ of the malaria belt and reports monthly malaria statistics for >7,000 health districts. The surveillance system uses a 40-item questionnaire that includes items concerning patient demographics, diagnosis, and area of residence (*14*). The spatial, temporal, and overall quality of this surveillance program, combined with spatial mapping, presents an opportunity to identify ecologic risk factors within an extensive existing surveillance network. Our hypothesis was that deforestation is positively associated with higher malaria risk in health districts in Mâncio Lima, Acre State, Brazil (Figures 1, 2). We also examined the association of 2006 malaria incidence with socioeconomic and demographic factors, including age, access to care, method of surveillance, sex, and malaria type.

**Figure 1:**
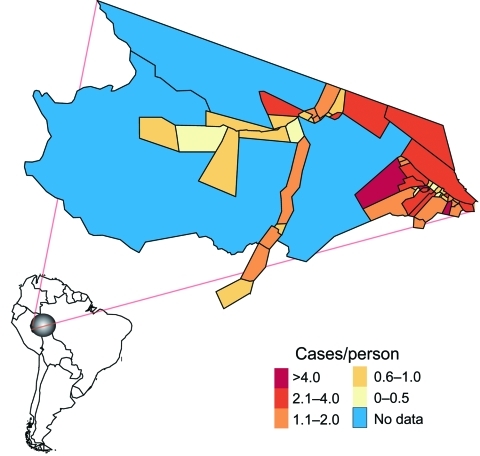
Mâncio Lima is the westernmost county in Brazil. The 2006 malaria incidence (cases/person) is mapped according to health districts (n = 54).

**Figure 2:**
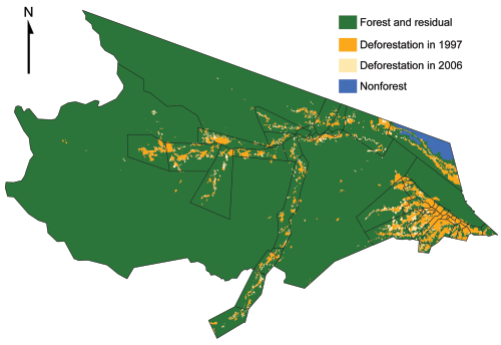
Deforestation trends in Mâncio Lima, Brazil, based on PRODES (Programa de Cálculo do Desflorestamento da Amazônia) 60 × 60–meter classified satellite imagery. The health districts are outlined in black. Baseline deforestation that occurred in 1997 is orange, deforestation that occurred between 1997 and 2006 is light brown, nonforested land is blue, and forested land is green.

## Materials and Methods

### Study Area

Mâncio Lima (4,672 km^2^) is situated in Acre State and is the westernmost county in Brazil, sharing a border with Peru to the west and Amazonas State to the north. Between 2000 and 2008, the population of the county increased 30% from 11,095 to 14,387. The county has 4% more men than women and a mixture of rural (48%) and urban (52%) households (*15*). The 67% of the territory that is considered uninhabited is made up of the Nukini and Poyanawa Indigenous Reserves and a portion of the Serra do Divisor National Park. The rural economy is based on agriculture and manioc flour production, and no areas have been licensed for mining exploration (*16*–*18*). Mâncio Lima has an average 4–30 cm monthly precipitation range and 19°C–32°C average monthly temperature range (*19*). The city of Mâncio Lima, which is the administrative and main population center, is connected by highway to Curzeiro do Sul, 24 km to the east. In 2006, Cruzeiro do Sul and Mâncio Lima ranked second and fourth highest, respectively, for malaria risk, and combined they reported 12.5% of all malaria cases in Brazil (*20*).

### Health Data

Since 2003, PNCM has practiced a malaria control strategy that reports all suspected malaria cases, identified from both slide-confirmed passive and active surveillance, for local health districts, which are often points of care. For each case, the survey tool records date, age (<10 years), sex, whether or not care was received within 48 hours of symptom onset, malaria type (*Plasmodium vivax* or *P. falciparum*; we classified mixed infections as falciparum), and method of surveillance (passive or active). In addition, the malaria case report form includes voluntary questions on education level and occupation type (*14*). We screened for patients who were residents of the health district in which they sought treatment and extracted monthly and annual percentages of these records from the Information System of Epidemiologic Surveillance of Malaria (SIVEP MALÁRIA) for the county of Mâncio Lima using Tableau 4.0 (www.tableausoftware.com) and Excel version 11.3 (Microsoft Corp., Redmond, WA, USA).

### Remote Sensing

In 2006, health district boundaries in Mâncio Lima were initially drawn by health district field staff and then mapped in real time with a GPS Garmin 12XL (Garmin International, Inc., Olathe, KS, USA). Then Track Maker 13.0 (www.gpstm.com) and ArcView 3.2 (www.esri.com) software were used to convert the paths into 54 health district polygons, and the population of each health district was enumerated. Next, the geographic data of each health district was linked to SIVEP MALÁRIA data. The uninhabited portion of the county, including the indigenous reserves and the national park, was divided into 3 geographic areas and excluded from data analysis (*21*).

Classified deforestation estimates at 60 × 60–meter resolution from 1997, and 2000 through 2006 were downloaded from the Programa de Cálculo do Desflorestamento da Amazônia (PRODES) in the National Institute for Space Research (*22*) (Figure 2). PRODES processes photographic images and Landsat imagery acquired at 30 × 30–meter resolution and is considered the gold standard reference for spatial deforestation data (*23*,*24*). The classification of deforestation in PRODES is cumulative; once a unit is deforested, it does not revert back to forest (*25*). Subsequently, in our analysis, we do not consider the effects of regrowth. ArcMap version 9.3 (www.esri.com) was used to calculate the health district geometric center and the amount of deforestation observed in 1997 and during 2000–2006.

### Analysis and Modeling

Summary statistics of variables from the SIVEP-MALÂRIA database for 2006 and deforestation data were calculated and mapped according to the geographic boundaries of the health districts. Numbers of malaria cases in 2006 for each health district were modeled by using a negative binomial (overdispersed Poisson) generalized additive regression model with a log-link function. For each health district, (log) census population for 2006 was included in the model as an offset term. Geographic location (latitude/longitude) was included in all models as a penalized 2-dimensional thin plate regression spline with smoothing parameter chosen by generalized cross-validation (*26*). Initial models considered deforestation and social or demographic variables to the above model individually.

A multivariable model was constructed in a stepwise fashion based on optimizing Akaike information criterion (AIC) (*27*). As before, the dependent variable was the number of malaria cases in 2006 for each health district and (log) census population for 2006 was included as an offset term. Deforestation variables of interest included absolute deforestation in 1997, absolute deforestation in 2006, percentage of deforestation in 1997, percentage of deforestation in 2006, and percentage change in deforestation during 1997–2006, 1997–2005, 1997–2004, 1997–2003, 1997–2002, 1997–2001, 1997–2000, 2001–2006, 2002–2006, 2003–2006, 2004–2006, and 2005–2006. Percentage of deforestation change was calculated by obtaining the percentage difference of deforestation in each health district between 2 time points. Social and demographic risk factors included percentage of malaria cases detected through active surveillance, percentage of malaria patients who received access to care in <48 hours of symptom onset, percentage of malaria patients <10 years of age, percentage of malaria patients who are male, and percentage of falciparum malaria cases. Interactions between variables selected as main effects in the stepwise model were also considered. Relative risks are presented for a ±1 SD change in the risk factor. Residual spatial autocorrelation was assessed by using Moran I with k = 4 distance-based neighbors. A 2-sided p value of 0.05 was considered to be statistically significant. Maps, statistical analysis, and figures were completed in R version 2.9.2 and Adobe Illustrator version 10.0.3 (www.adobe.com) (*28*).

## Results

**Figure 3:**
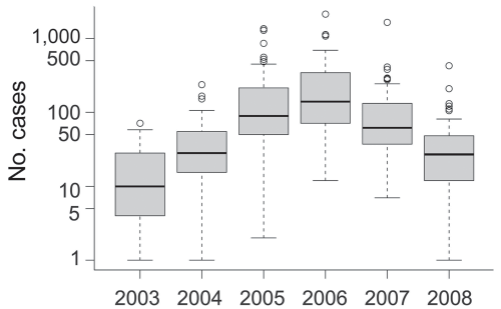
Box-and-whisker plots of slide-confirmed malaria cases on a logarithmic scale by health districts in Mâncio Lima, Brazil, 2003–2008. Error bars indicate interquartile ranges, and thick horizontal bars indicate the median.

**Figure 4:**
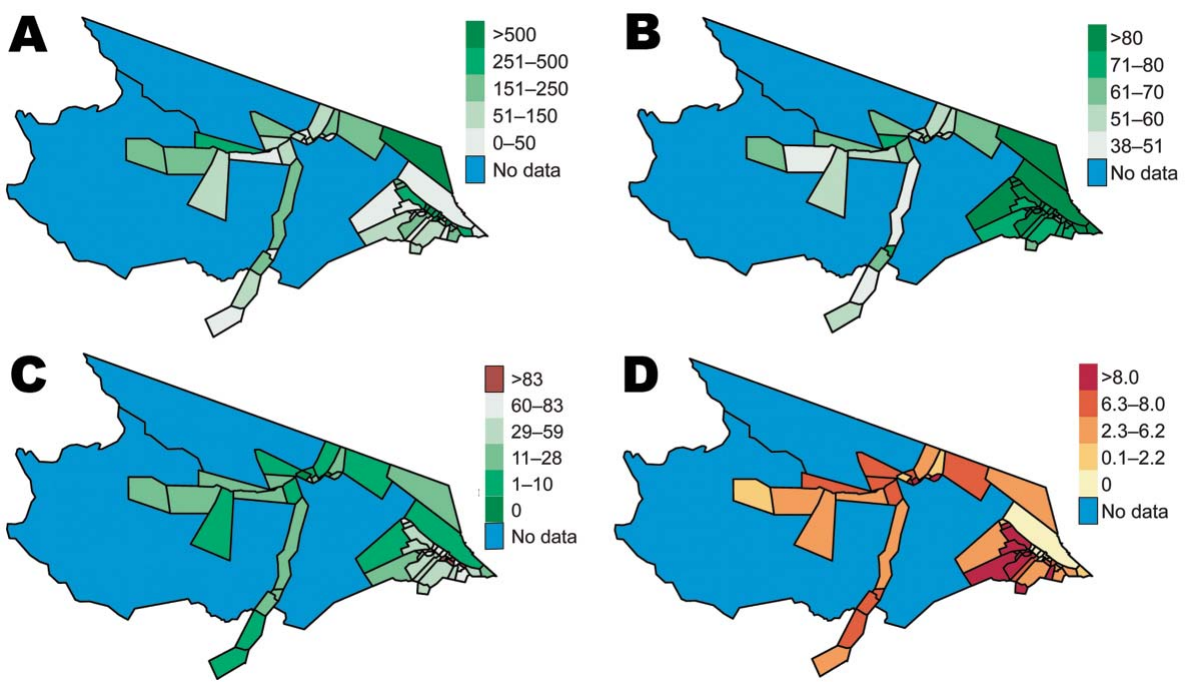
Cloropleth maps of selected malaria risk factors for health districts in Mâncio Lima, Brazil. A) Resident population in health districts in 2006. B) Percentage of slide-confirmed malaria cases receiving access to care within the first 48 hours of symptom onset in 2006. C) Percentage of 1997 deforestation in each of the health districts calculated from 60 × 60-meter resolution classified PRODES data. D) Cumulative percentage change in deforestation by health district from 1997 to 2006. Uninhabited areas are excluded from the analysis.

Fifty-four health districts occupy 27% (1,270/4,760 km^2^) of Mâncio Lima and spatially reflect the population settlements along 2 dominant river channels and in the urban zone around the city of Mâncio Lima. In 2006, the health districts reported a total of 15,437 slide-confirmed malaria cases, a mixture of both falciparum (41%) and vivax (59%) malaria. Most malaria patients across health districts were males (56%) >10 years of age (72%); the cases were identified by active surveillance (65%), and the patients received access to care within 48 hours of symptom onset (71%). The average incidence rate of the malaria epidemic was 1.16 cases/person, but within individual districts, the incidence was 0.4–12 cases/person (Figures 1, 3). We were unable to analyze answers to categorical questions on the education level and activities of case-patient activities within the previous 2 weeks because the response rates were insufficient. Choropleth maps depicting population distribution, access to care, malaria incidence, and percentage of deforestation change during 1996–2006 are shown in Figure 4.

Baseline deforestation in 1997 was concentrated within and near the city of Mâncio Lima, with varying degrees of deforestation found in the health districts along the river ways. The most deforestation change between 1997 and 2006 was located just west and south of the city. Over this period, percentage of deforestation in health districts increased, on average, 6.6%–26%. The SD of this increase is 5.9% (Table). Notably, a large wetland area northeast of the city limited the amount of land clearing taking place in that area (Figures 2,4).

**Table:** Influence of ecologic deforestation and social demographic risk factors on malaria incidence in health districts, Mâncio Lima County, Brazil, 1997–2006*

Variable	2006 health district summary			Univariate analysis†				Multivariate analysis†	
	Mean	SD		RR	95% CI	AIC		RR	95% CI
Ecologic									
Deforested in 1997, %	40.2	32.5		0.84	0.64–1.09	671.1			
Deforested 1997–2002, %	3.2	4.3		1.31	1.11–1.56	664.0			
Deforested 1997–2001, %	2.7	4.2		1.32	1.11–1.57	663.8			
Deforested 1997–2000, %	2.3	4.3		1.33	1.12–1.58	663.6		1.48	1.26–1.75
Deforested 2001–2006, %	3.4	3.6		1.03	0.85–1.23	672.7			
Social demographic									
Active surveillance, %	64.9	19.3		1.27	0.97–1.65	669.6			
Access to care <48 h, %	70.6	13.5		1.18	0.87–1.59	671.4		0.92	0.72–1.17
Case-patients <10 y, %	27.6	9.3		1.18	0.94–1.46	669.9			
Case-patients, male, %	55.9	7.8		1.07	0.88–1.31	672.1			
Falciparum cases, %	41.3	10.1		1.11	0.87–1.41	671.0			
Spatial									
Area, km^2^	23.9	33		1.20	0.99–1.46	669.1		1.26	1.06–1.49
Interaction‡								1.20	1.05–1.39

*Summary statistics of variables, relative risks (RR), and 95% confidence intervals (CIs) for the univariate and multivariate negative binomial generalized additive models with integrated smoothness estimation of spatial correlation. The SD is used as the unit of analysis for all risk factors and the Akaike information criterion (AIC) values for the univariate models are shown. The AIC for the multivariate model is 655.9. †Models adjusted for spatial trend ‡Area × access to care.

The univariate analysis adjusts for variability between the health districts, and spatial trend. We show the influence of ecologic deforestation and social demographic risk factors on malaria incidence. Percentage of deforestation during 1997–2000 is the factor most predictive of malaria risk in the health districts on the basis of model AIC. Health districts that deforested 4.3% (±1 SD) from 1997 to 2000 are associated with 1.33 (95% confidence interval [CI] 1.12–1.58) increase in malaria risk. Historic baseline deforestation in 1997 is not significant, but malaria risk and percentage deforestation from 1997–2002, 1997–2001, and 1997–2000 are significant and positively correlated. More recent percentage deforestation changes from 2001 through 2006 are not associated with malaria risk, along with 1997 and 2000–2006 measures of absolute deforestation and cumulative percent deforestation (Table).

Although these results were not quite significant in the univariate analysis, the risk of malaria is 1.27 (95% CI 0.97–1.66) when active surveillance increases by 19% within a health district. Malaria risk is 1.18 (95% CI 0.87–1.59) when 14% of more cases obtain care within the first 48 hours of symptoms. The spatial size of health districts is also nearly significant as the relative malaria risk is 1.20 (95% CI 0.97–1.48) for a 32 km^2^ increase in health district size. Associations with malaria risk based on age, sex, or malaria type are not significant (Table).

**Figure 5:**
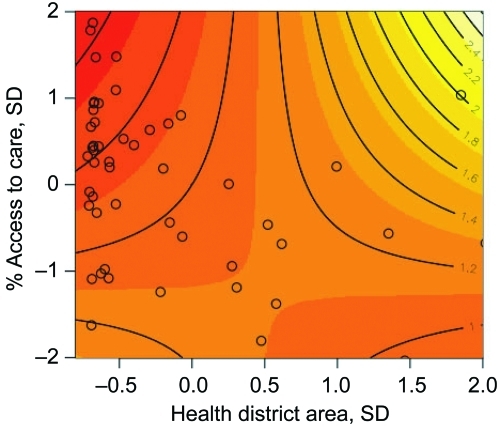
Joint relative risk plot of access to care and health district spatial area, Mâncio Lima, Brazil. Contour lines indicate the joint relative risk for standard deviation changes in percentage access to care and health district spatial area. Open circles are the observed percentage access to care and health district spatial area size data pairs for the 54 health districts. The contour line increment of relative risk is 0.2, increasing with the shading from red to white.

**Figure 6:**
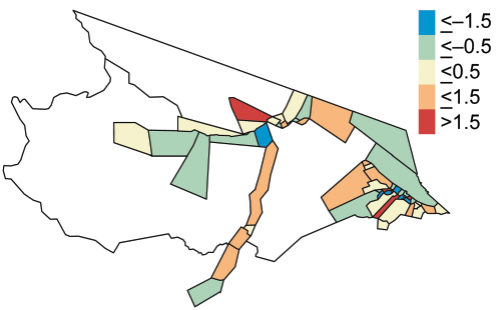
Multivariate model residual map, Mâncio Lima, Brazil.

The multivariate analysis shows a 4.3% increase in the percentage of deforestation between 1997 and 2000 is associated with a malaria risk of 1.48 (95% CI 1.26–1.75) after access to care and the spatial area of the health districts are adjusted for (Table). Figure 5 shows the interaction and joint relative risk of percentage access to care and the spatial area on malaria incidence within each health district adjusted for percentage deforestation from 1997 through 2000. In Mâncio Lima, malaria risk decreases as the percentage of access to healthcare increases for health districts <23.9 km^2^ (mean value). The pattern of relative risk in health districts of larger size is less clear, due to a shortage of observations. A map of the model residuals did not show any spatial trends; the global spatial autocorrelation of the residuals based on Moran’s I is –0.12 and not significant (p = 0.90) (Figure 6).

## Discussion

We based our investigation of environmental and sociodemographic malaria risk factors on an existing surveillance system and estimated the relative risk for these factors at the health district level. Malaria surveillance in Brazil is unprecedented in scale and uniformity. Focusing on 1 county linked to global information systems health district level data, we report the characteristics of the health districts, map the distribution of risk factors, and find significant associations between deforestation and malaria incidence. Adjusting for population, access to care, and district size, we found that malaria risk increased ≈50% in health districts when 4% of the area underwent deforestation in 1997–2000.

Our approach shows the relative associations of malaria incidence and deforestation patterns across space, rather than a trend of malaria incidence and deforestation across time. The model assumes annual regional variability in temporal risk factors, such as climate and intervention measures, was uniform for the 2006 observations. We examined measures of deforestation before 2006 to model the pattern of malaria observed in 2006. However, given the cross sectional design, the association of malaria incidence to prior deforestation does not necessarily imply a causal trajectory of increased deforestation and elevated malaria incidence.

We found that the univariate models predict that a higher malaria risk is associated with more active surveillance and access to care. This seems counterintuitive, as active surveillance generally identifies cases quickly, which leads to faster treatment and lower disease risk, and access to care is a variable reliably associated with lower disease rates. For example, in the Indian state of Assam, malaria incidence is consistently lower in villages within 5 km of healthcare facilities (*29*). The univariate relative risks suggest that increased active surveillance and access to healthcare during the epidemic led to the identification of cases that normally would have gone unreported. The significant interaction of health district size and access to care improved the performance of the multivariate model for percentage of deforestation from 1997 through 2000, health district size, and access to care. The interaction and joint relative risk show that increased surveillance in health districts <23 km^2^ is protective against malaria risk, after adjusting for percentage of deforestation.

The landscape establishes local ecology and biodiversity, and our results confirm that cleared land is associated with a higher malaria risk. This association has been identified in previous research, but here we link the ecologic observations of the habitat preference of *A. darlingi* mosquitoes for deforested areas to an existing malaria surveillance program. Moreover, we found that human malaria risk is specifically associated with deforestation 5–10 years previously. We did not find an association with deforestation before or after that time frame. These findings seem to agree with other research that observed that shrub land cover, which develops 5 years after deforestation and becomes classified as secondary growth ≈15 years after deforestation, has significantly greater abundance of *A. darlingi* larvae than does forested land (*8*). Together these findings suggest that entomologic risk is based on the fate of cleared land.

The study is limited by several factors. The malaria data are based on annual percentage measures derived from the PNCM malaria surveillance questionnaire. Each health district was the unit of analysis, so we were unable to adjust for risk factors at the individual case level. The categorical data structure was restrictive and there was insufficient reporting on voluntary portions of the survey that limited our ability to adjust for socioeconomic drivers. The frequency of double reporting is unknown, but we have filtered the data for only those patients who reported living within the health district where they sought treatment. Temperature is nearly always suitable for malaria transmission in the Amazon Basin, but any variability of rainfall and hydrologic characteristics in Mâncio Lima may also be a confounding factor (*30*).

Another consideration is the absence of current immigration information, but several observations suggest that migration is an unlikely factor in explaining malaria patterning in this study site. In 2006 there were a total of 750,000 emigrants living in the Northern Region, which encompasses the states of Acre, Amazonas, Anapá, Roraima, Rondônia, Pará, and Tocantins. This represents just 4% of all emigrants to new regions within Brazil based on place of birth. More locally in 2000, Máncio Lima recorded an influx of just 29 emigrants >5 years of age since 1995 from areas outside of Acre, or just 0.2% of all migration to Acre (*15*). These trends suggest that a minimal amount of migration to Mâncio Lima occurred before 2006.

The emerging local aquaculture industry is an important concern that might also be correlated with the deforestation patterns in health districts. Pond, wells, or fish farms >50 m in circumference significantly increase the abundance of *A. darlingi* larvae (*9*). Mâncio Lima's aquaculture production has been growing since this association was established in 2003. The ponds ranged size from 5 ha to 175 ha, and the April 2007 harvest yielded 30,000 kg of fish, valued at R$216,000 or US$106,000 (*31*). Taking the 2007 harvest at a yield obtained in the neighboring state of Amazônia of ≈70 kg/ha/year, suggests that ≈430 ha of aquaculture existed in Mâncio Lima in 2006 (*32*). Fish farms are often located in degraded and deforested lands, yet this practice maybe leading to more mosquito larval habitat and higher malaria incidence. Further investigation is needed to differentiate deforestation from the effects of fish farming.

Our models assume that environmental exposures occurred in the health district in which a patient claims residency. If persons slept and worked in different areas, we could not directly associate exposure with environmental variables within the health district. However, the diurnal biting pattern of *A. darling* mosquitoes, which generally peaks in the evening and sometimes in the early morning, means most exposure will occur near the home (*7*,*10*,*33*). Additionally, we were not able to adjust for the presence or absence of the agent, plasmodium sporozoites, yet the county was saturated with malaria at the peak of an epidemic, which increased the probability of widespread malaria exposure. In a scenario in which *A. darlingi* mosquitoes are very abundant but the parasite is absent, once the malaria sporozoite is introduced, malaria should spread.

We showed how the framework of health districts can link landscape and disease risk, but the overall generalizability of our findings is limited. In the Amazon, patterns of malaria risk factors are known to change from 1 community to the next. We found that age and sex were not associated with malaria risk in Mâncio Lima, yet men carry double the risk of women in some communities, and in others, gold miners have a risk 3 times higher than that of urban residents (*34*). Another community has no age-specific, occupational, or gender risks, but activities such as strolling outdoors after 6:00 pm and waking before 6:00 am for adults, and attending church services in the evening for children are significantly associated with malaria risk (*35*). Beyond the Amazon, evidence has shown that mosquito survival can depend on slight variations in temperatures, humidity, and sunlight as a result of deforestation (*36*,*37*). Even though eradication of malaria is a reemerging priority of the global health community, no spatially standardized approach has been developed that can monitor patterns of malaria at the clinic or treatment unit (*38*).

At present, policy makers and epidemiologists continue to speculate about the regional and local variation of malaria and malaria risk factors. But policy makers also know that, “policies are sometimes applied more broadly than appropriate to large regions when it may actually only be relevant to a particular setting within the region … (and) policies often need to be specific to be useful” (*38*, p. 95). Currently, beyond our findings in Mâncio Lima and a few isolated studies, the ultimate relationship and geographic extent of the malaria incidence and deforestation process are unknown.

Our findings illustrate the importance of relative deforestation between health districts in the county of Mâncio Lima, but they do not necessarily explain the overall intensity of the 2006 epidemic (Figure 3). The epidemic is likely a result of a combination of forces with deforestation (and perhaps aquaculture) creating the landscape conditions more suitable to *A. darlingi* mosquitoes. The extensive drought of 2005 may also have contributed to higher mosquito populations and malaria risk (*39*). This conclusion is supported by studies of El Niño events, caused by warming sea surface temperatures, which decreased precipitation in the region and are associated with higher malaria incidence the following year in Venezuela and Guyana (*40*). Here we show that deforestation significantly affects malaria risk, which suggests that land use measures may be 1 method to employ in malaria control.

In summary, we show that focused monitoring and high resolution spatial mapping of health districts can identify ecologic associations between malaria incidence and deforestation. Other human health and ecology linkages may be discernable with similar high resolution and spatially explicit data.
